# Altered BMP2/4 Signaling in Stem Cells and Their Niche: Different Cancers but Similar Mechanisms, the Example of Myeloid Leukemia and Breast Cancer

**DOI:** 10.3389/fcell.2021.787989

**Published:** 2022-01-03

**Authors:** Boris Guyot, Sylvain Lefort, Thibault Voeltzel, Eve-Isabelle Pécheur, Véronique Maguer-Satta

**Affiliations:** ^1^ CNRS UMR5286, Centre de Recherche en Cancérologie de Lyon, Lyon, France; ^2^ Inserm U1052, Centre de Recherche en Cancérologie de Lyon, Lyon, France; ^3^ Université de Lyon, Lyon, France; ^4^ Department of Cancer Initiation and Tumor Cell Identity, Lyon, France; ^5^ Université de Lyon 1, Lyon, France; ^6^ Centre Leon Bérard, Lyon, France

**Keywords:** BMP, stem cells, cancer, microenvironment, mesenchymal, bisphenol, environmental exposure, resistance

## Abstract

Understanding mechanisms of cancer development is mandatory for disease prevention and management. In healthy tissue, the microenvironment or niche governs stem cell fate by regulating the availability of soluble molecules, cell-cell contacts, cell-matrix interactions, and physical constraints. Gaining insight into the biology of the stem cell microenvironment is of utmost importance, since it plays a role at all stages of tumorigenesis, from (stem) cell transformation to tumor escape. In this context, BMPs (Bone Morphogenetic Proteins), are key mediators of stem cell regulation in both embryonic and adult organs such as hematopoietic, neural and epithelial tissues. BMPs directly regulate the niche and stem cells residing within. Among them, BMP2 and BMP4 emerged as master regulators of normal and tumorigenic processes. Recently, a number of studies unraveled important mechanisms that sustain cell transformation related to dysregulations of the BMP pathway in stem cells and their niche (including exposure to pollutants such as bisphenols). Furthermore, a direct link between BMP2/BMP4 binding to BMP type 1 receptors and the emergence and expansion of cancer stem cells was unveiled. In addition, a chronic exposure of normal stem cells to abnormal BMP signals contributes to the emergence of cancer stem cells, or to disease progression independently of the initial transforming event. In this review, we will illustrate how the regulation of stem cells and their microenvironment becomes dysfunctional in cancer via the hijacking of BMP signaling with main examples in myeloid leukemia and breast cancers.

## Introduction

Cancer is a major public health issue considering its high mortality rate, increasing incidence, and cost to society. Despite tremendous progress in the development of targeted therapies, most cancers relapse owing to cancer stem cell (CSC) survival and treatment escape (Clarke, 2019). Two major axes remain to be solved to decrease the impact of cancer, one is to identify early and reliable signs of tumor onset to prevent further transformation, taking into account the origin and properties of the niche, the other is to counteract CSC resistance before tumor progression and relapse (Saygin et al., 2019). The existence of spatially defined areas (called niches) essential for stem cell (SC) maintenance in adults was demonstrated in the bone marrow, and later in epithelial tissues and cancers. The microenvironment is a dynamic and complex milieu critical for the delivery of signals orchestrating cell proliferation, differentiation and apoptosis. Following the identification of different subsets of SCs, the composition and functions of adult SC niches began to be elucidated in the hematopoietic system and in solid tissues (Batsivari et al., 2020). Mesenchymal stromal cells (MSCs), a population of long-lived stem/progenitor cells, that contribute to cellular diversity and architecture of the niche and which play a central role in growth, survival and resistance of SCs and tumor cells. MSCs also secrete many morphogens, growth factors and cytokines, including BMPs. The definition of the tumor niche *per se* is still unclear, as its properties related to cancer onset, evolution and resistance have not been identified. Main hypotheses suggest that CSC resistance reflect the preservation of intrinsic protective mechanisms unique to the SC compartment or the re-emergence of these properties in cancer cells (O'Brien-Ball and Biddle, 2017). In addition, CSCs and their niche are engaged in a crosstalk regulating several SC-signaling pathways, as well as niche features (Arora and Pal, 2021). BMP2 and BMP4, produced within the SC microenvironment, are master regulators of the functions of tissue-specific SC and their surrounding MSC and emerge as key players of SC transformation. Here, we will focus on the role of BMP2 and BMP4 signaling in human hematopoietic and breast epithelial SC regulation, transformation, maintenance and drug resistance, in association with their microenvironment.

### BMP2 and BMP4 Have Distinct Effects in Human Stem Cells

Hematopoiesis is supported by hematopoietic SCs (HSCs) and controlled by soluble factors, as well as cell/cell and cell/ECM interactions. Alterations of these processes induce various pathologies including leukemia that can directly affect HSCs. The influence of TGFβ, BMP and activin signaling on human HSCs can be investigated by studying regulators of the follistatin family, such as FLRG (FoLlistatin Related Gene). FLRG interacts with several members of the TGFβ family (Activin A and BMP2, BMP4, BMP6 or BMP7) (Tsuchida et al., 2000; Tortoriello et al., 2001; Maguer-Satta et al., 2003), and regulates HSCs during hematopoietic differentiation (Maguer-Satta et al., 2001). BMP2 fosters commitment of human HSCs toward erythroid cells, whereas BMP4 controls HSC self-renewal or megakaryocytic lineage engagement. Both FLRG and follistatin regulate erythroid commitment of human HSCs induced by activin or BMP2 (Maguer-Satta et al., 2003). Additionally, FLRG and follistatin molecules binding to the fibronectin type I domains of the fibronectin protein (outside of the integrin β1 binding domains) regulates HSCs by inducing their adhesion to fibronectin (Maguer-Satta and Rimokh, 2004; Maguer-Satta et al., 2006). This illustrates how BMP signaling can regulate HSCs by modulating their interaction with the microenvironment. Unlike BMP2, a strong cooperation between BMP4 and other cytokines related to HSC maintenance and megakaryopoiesis, such as Stem Cell factor or thrombopoietin, was identified. In the absence of thrombopoietin, BMP4 induces HSC commitment toward the megakaryocytic lineage, as well as terminal differentiation, leading to platelet production. BMP4 also induces a higher level of adhesion of human HSCs and progenitors to fibronectin than thrombopoietin (Jeanpierre et al., 2008). The importance of BMP4 in controlling HSC functions, through alpha4 integrin-mediated adhesion, was further documented in a murine model (Khurana et al., 2013). Therefore, BMP2 and BMP4 appear to play different regulating roles on human HSCs, whereas their function in murine hematopoiesis was reported to be more redundant (Bhatia et al., 1999; Borges et al., 2013; Khurana et al., 2013; Singbrant et al., 2020).

Epithelial cells and cells of the normal mammary gland environment (fibroblasts, adipocytes, hematopoietic cells) produce BMP2 and BMP4, suggesting a role in mammary SC regulation (Chapellier et al., 2015). The function of BMPs in normal breast was explored by isolating primary human epithelial cells, SCs and progenitors. Immature epithelial cells (SCs and progenitors) express different elements of the BMP pathway, indicating that BMPs could play a role in normal SC regulation. As for HSCs, BMP2, and BMP4 have distinct effects on SC regulation. Whereas BMP4 modulates the SC compartment and myoepithelial progenitors, BMP2 fuels commitment and proliferation of luminal progenitors (Chapellier et al., 2015; Clement et al., 2017). This is consistent with results reported for mammary gland development in mice, showing that BMP2 is involved in the regulation of the luminal lineage (Forsman et al., 2013). Despite their similarities, BMP2 and BMP4 thus exert different functions on human SCs in the mammary gland and hematopoietic system. These data indicate that BMP2 might preferentially affect lineage-committed progenitors, whereas BMP4 may have a broader effect on SCs and less tissue specific cells (megakaryocytic or myoepithelial progenitors). Likewise, BMPR1b and BMPR1a play distinct roles in MSCs fate regulation. Unlike BMPR1b, BMPR1a initiates both osteogenesis and chondrogenesis (Kaps et al., 2004). Conversely, alteration of BMPR1b expression in MSCs has been reported to reduce the bone mass and alter their osteo-differentiation (Shi et al., 2016). Despite these controversial data, it appears that BMPR1a and BMPR1b pathways, as well as those implying BMP2 and BMP4, are likely distinct and may not substitute to each other even if their respective roles in various SC cell fate remains unclear.

### Abnormally Persistent BMP Signaling Initiates Transformation or Reprogramming Toward a SC-like Phenotype

Abnormalities of the BMP pathway have been reported at advanced stages of various cancers. Recent investigations reported its importance in early transformation steps of hematopoietic and breast tissues. Chronic Myeloid Leukemia (CML) represents the reference model for SC transformation, whereas breast tumors contain CSC of debated origin. CML arises from a SC transformation event, induced by a single translocation generating the BCR-ABL oncogene. Patient samples at diagnosis revealed a dysregulation of several actors of the BMP pathway during chronic phase of the disease, with clear differences between mature (CD34^−^) and immature (CD34^+^) compartments (Laperrousaz et al., 2013). Alteration of BMP receptor type 1b (BMPR1b) expression at the HSC surface is induced by the expression of BCR-ABL and these molecular changes led to altered responses of leukemia cells to BMP2 and BMP4 as compared to normal bone marrow cells. Leukemic BMPR1b^high^ cells respond to BMP4 by amplifying and maintaining their CSCs population, whereas BMP2 favors the expansion of myeloid CML progenitors. A similar role for BMP2 in the maintenance of CSCs was recently reported in hepatocellular carcinoma (Guo et al., 2021). In addition, BMPR1b mutations were linked to Brachydactyly type A1 characterized by bones hyperplasia, indicating that BMPR1b alterations could affect both the HSCs and MSCs in different physiopathological contexts by affecting their response to BMP4 (Laperrousaz et al., 2013; Racacho et al., 2015). In CML, dysregulation of intracellular BMP signaling mediated by BCR-ABL corrupts and amplifies the response to exogenous morphogens released by the niche, which are abnormally abundant and directly influence CSC fate. Indeed, concomitantly to intrinsic SC alterations, an increase in soluble BMP2 and BMP4, compared to healthy individuals, was detected within the tumor microenvironment of leukemia (Zylbersztejn et al., 2018) and breast cancer (Chapellier et al., 2015). Abnormal BMP2 production by SCs microenvironment together with BMP2-driven alterations of epithelial SC fate are involved in the emergence of luminal breast cancer cells (Chapellier et al., 2015). Chronic exposure of human immature mammary cells to high BMP2 levels initiates SC transformation toward a luminal tumor phenotype. Dysregulation of BMPs within the SC niche could then promote early steps of luminal transformation of resident epithelial cells through the following sequence of events: BMP2 binds to BMPR1b, and change the transcription factor balance FOXA1/FOXC1 in favor of FOXA1, concomitant to an upregulation of GATA3. Transformation then proceeded from an aberrant amplification of the natural response to BMP2, driving SC commitment toward luminal lineage and further expansion of luminal progenitors (Chapellier et al., 2015; Clement et al., 2017). A similar mechanism was observed in ovarian cancer (Choi et al., 2015). In breast and hematopoietic cancers, these data demonstrate that niche-secreted BMP2/BMP4 promote SC transformation through the amplification of a normal SC response, linked to their chronic exposure to high levels of these morphogens (Chapellier et al., 2015; Clement et al., 2017). Importantly, the presence of an inflammatory signal (such as IL6) appeared to be important to stabilize and maintain the transformed phenotype (Reynaud et al., 2011; Chapellier et al., 2015). Previous studies have shown that up-regulation of BMP4 could also be involved in transformation initiation in an inflammatory context, for example by promoting a metaplastic condition in which normal squamous esophageal epithelium is replaced by columnar epithelium (Barrett esophagus) (Milano et al., 2007). Altogether, these data also highlight the ability of the SC niche to deliver cues dictating the tumor phenotype.

Another mechanism to generate cancer stem cells could emerge from a “reprograming” process (O'Brien-Ball and Biddle, 2017) and results from a continuous exposure of mature cells to BMP2 and BMP4. Notably, BMP2 and BMP4 are overexpressed in the bone marrow of Acute Myeloid Leukemia (AML) patients (Voeltzel et al., 2018), supporting the aggressive clonal malignancy through excessive proliferation of immature cells blocked in their differentiation process. At the molecular level, alterations of the BMP pathway favor survival of chemotherapy-resistant immature-like leukemic cells. Binding of BMP4 to BMPR1a leads to ΔNp73 expression in mature AML blast cells, which in turn induces Nanog, reminiscent of cell “reprogramming” toward a CSC-like phenotype (Voeltzel et al., 2018). These features are associated with poor patient prognosis and treatment response. This was the first demonstration of a niche-driven AML leukemia cell reprogramming toward an SC-like phenotype, featuring BMP4 as a key stemness regulator. Interestingly, in many other tissues like brain, gastro-intestinal tissues, colon or liver, the importance of BMP4 and BMPR1a to predispose, initiate or maintain stemness of transformed cells was also evidenced (Brosens et al., 2011; Hover et al., 2016; Wang et al., 2019; Schwarzmueller et al., 2020). Nevertheless, for a given normal tissue, BMP signaling can display opposite functions such as preventing epithelial cells de-differentiation like in the intestinal murine tissue (Koppens et al., 2021).

**FIGURE 1:**
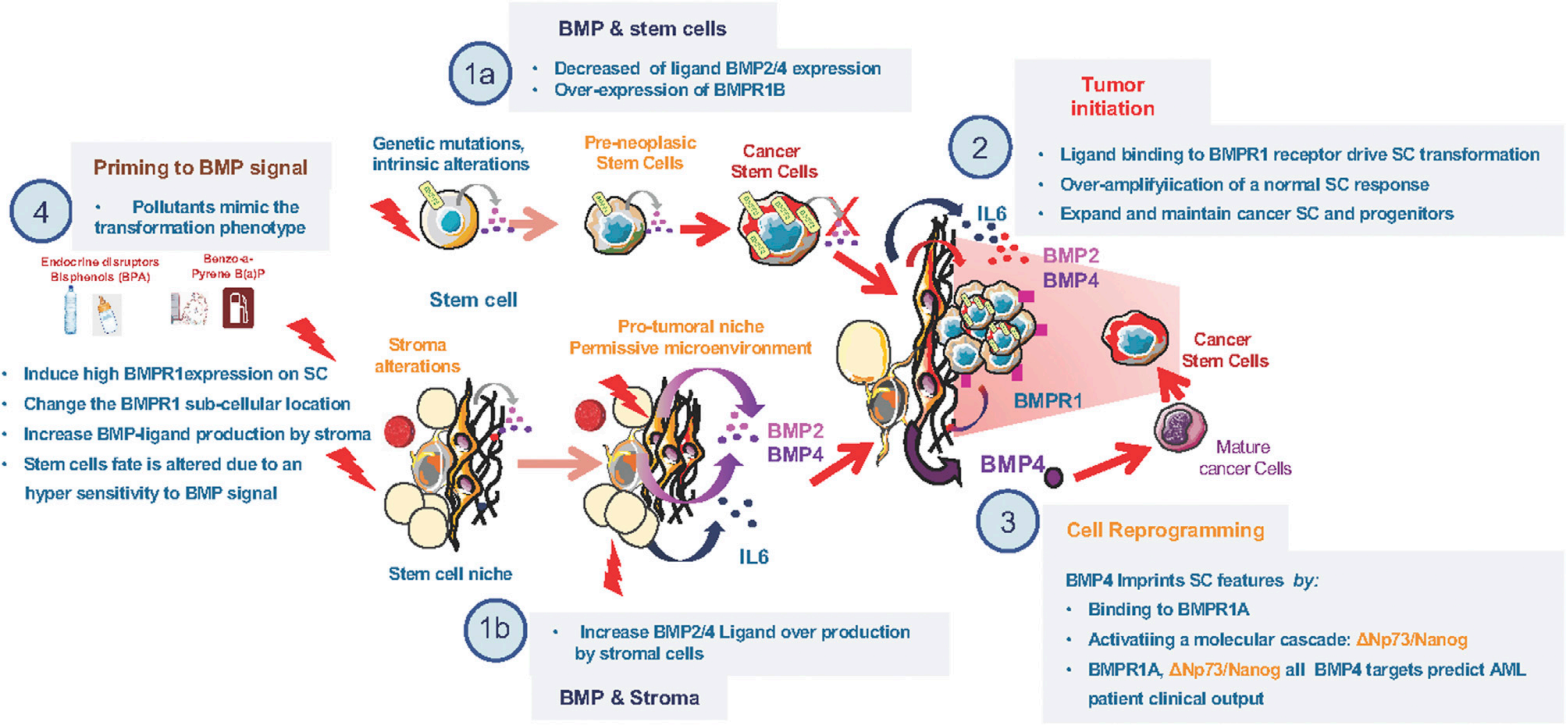
Alterations of the BMP pathway involved in early steps of transformation. **(1a)** Decrease in ligand (BMP2 and/or BMP4) and increase in type1 BMP-receptor at time of diagnosis in stem cells of diferent leukemia and breast cancer types. **(1b)** Increase in ligand (BMP2 and/or BMP4) production by stromal cells of the tumor microenvironement at time of diagnosis in diferent leukemia and breast cancer types. **(2)** Cooperation between BMP2 overproduction by the stroma and IL6 to induce BMPR1b overexpressing stem-cells transformation. **(3)** BMP4 mediated reprogrammation of mature cells toward an imature stem-like cell. **(4)** Effect of environmental polluant on the expression of both ligand and BMPR1 expression in healthy cells.

At diagnosis of leukemia and breast cancer, BMPR1b was evidenced as an important transducer of BMP signals, acting as an amplifier of the normal SC response to BMP (Figure 1). This likely contributes to CSC survival independently of the initial oncogenic event. It also reveals the importance of simultaneous intrinsic and extrinsic alterations of BMP signaling to fuel transformation and demonstrates the direct implication of in the emergence and expansion of CSCs by inducing an over-amplified and persistent response of SC to the BMP signal. Altogether, it provides a proof of the “seed and soil” concept in the context of BMP-driven cancers that require two complementary events, one taking place within the niche and the other directly acting on (stem) cells. Due to the broad involvement of BMP signaling in cancer, this mechanism might be common to different cancers, and sheds light on the involvement of BMPs in cancer cells stemness (Kim et al., 2015; Huang et al., 2017; Sachdeva et al., 2019; Sun et al., 2020).

### BMP Signaling as a Driver of Cancer Stem Cell Resistance to Treatment

**FIGURE 2:**
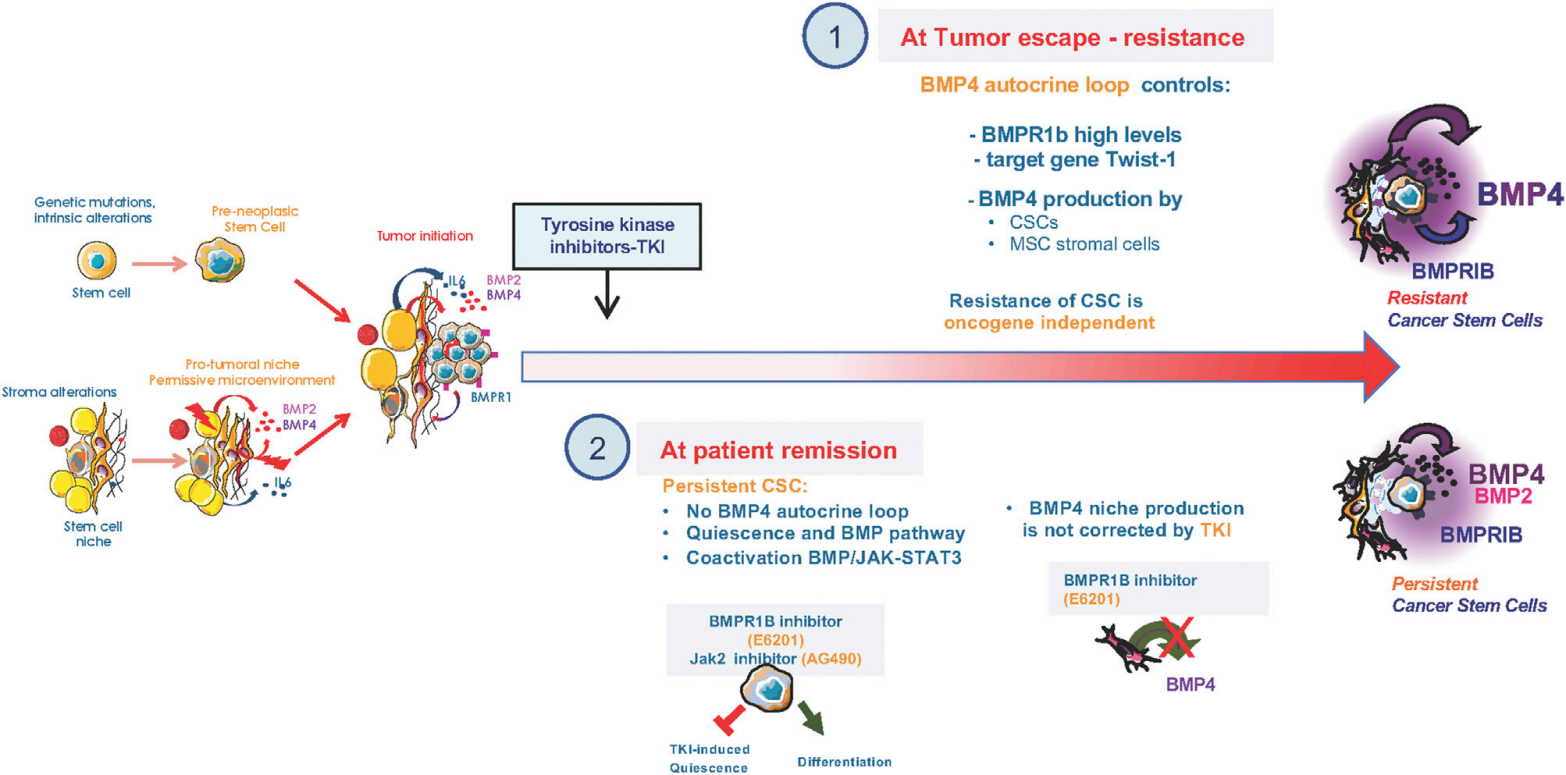
Alterations of the BMP pathway upon treatment of CML cancer stem cells. **(1)** Setting of a BMP4 autocrine loop in CSCs that controls Twist1 expression to promote resistance of CML CSCs to tyrosine kinase inhibitors (TKIs) and retained high production of BMP4 by bone marrow mesenchymal stromal cells of resistant patients. **(2)** At patients remission under TKI treatment, CSCs showed a co-enrichment in BMP and Jak2-signaling, quiescence and SC signatures, as identified by single cell RNA-Seq analysis of TKI-persisting cells. In persisting CSCs, BMPR1b-cells displayed co-activated Smad1/5/8 and Stat3 pathways that are targeted by blocking BMPR1B/Jak2 signal using, for example, a specific BMPR1b inhibitor (E6201) or Jak2 (AG490) inhibitor. The TKI-induced quiescence of residual CSCs relies on a BMP4 signal delivered by surrounding mesenchymal cells and inhibited by the BMPR1B inhibitor (E6201). Dual targeting of BMP and Jak2 efficiently reverses TKI-dependent induction of quiescence and allowed re-entry into a differentiation process of the BMPR1b^+^ CSC sub-fraction that was adherent to the stroma.

CSCs constitute a reservoir likely involved in cancer recurrence in many tumors as they resist to several treatments and sustain disease for years. In CML, the first anti-cancer targeted therapy was developed and paved the way to the family of Tyrosine Kinase Inhibitors (TKIs). It efficiently eliminates most cycling progenitors and has become the standard-of-care for CML. However, some CSCs remain treatment-insensitive and prolong disease recurrence for years leading to a proportion of patients that develop primary or secondary resistance to TKIs, of unknown mechanism in 30% of the cases. The embryonic gene *Twist-1,* a context-dependent target or a regulator of BMP signaling depending on the cellular context, is overexpressed in various tumors and associated with poor prognosis and resistance. In CML, *Twist-1* is an early predictive molecular marker of resistance to TKIs at diagnosis, especially in patients with unidentified resistance mechanisms (Cosset et al., 2011). Following long-term TKI treatment, CSCs persist and escape through an intrinsic BMP4 autocrine loop that induce Twist-1 expression (Grockowiak et al., 2017) and impacts on G1-S progression during cell cycle regulation (Savona and Talpaz, 2008; Toofan et al., 2018). Moreover, resistance to TKIs is accompanied by an further dysregulation of the CSC microenvironment, producing excessive BMP4 (Grockowiak et al., 2017; Jeanpierre et al., 2021). Consequently, many patients likely retain treatment-resistant CSCs within their primary tumor or secondary metastatic site. *De facto*, most therapies achieve remission but patients would relapse because of the re-activation of a quiescent/dormant clone in several cancers (Risson et al., 2020). The SC environment create a permissive niche for the emergence, survival, re-activation, and resistance against therapy-induced apoptosis of CSCs and has become an important target for anti-cancer therapy (Batsivari et al., 2020; Risson et al., 2020). CSCs include quiescent cells resistant to standard therapies, differing from their normal counterparts in both hematopoietic and solid tumors (White and Lowry, 2015; Batsivari et al., 2020). Several studies unraveled the importance of TGFβ/BMP signaling in SC dormancy and adaptation to treatment, in association with the tumor microenvironment (Risson et al., 2020; Nobre et al., 2021). In CML, single cell RNA-Seq analysis of TKI-resistant CSCs showed a co-enrichment of BMP and Jak2 signaling targets, quiescence and SC signatures (Jeanpierre et al., 2021). Using a new model of persisting CML CSCs, BMPR1b-expressing cells displayed co-activation of Smad1/5/8 and Stat3 pathways (Jeanpierre et al., 2021). Treatment-induced quiescence of residual CSCs relies on the activation of Jak2-Stat3 signaling, mediated by BMP4 released from surrounding mesenchymal cells (Jeanpierre et al., 2021). Targeting of BMPR1b and Jak2 efficiently reversed this TKI-induced quiescence of the BMPR1b^+^ CSCs adhering to the stroma, and allowed them to re-enter a differentiation process. Such a strategy may contribute to eliminate dormant CSCs. However, in brain tumors the role of the BMP signaling in CSCs resistance to radiotherapy or chemotherapy remains controversial. While BMP4 could efficiently directly reduce glioma stemness by inducing their differentiation and death (Nayak et al., 2020), other groups reported that BMP inhibition only reduce cell proliferation without affecting stemness properties (Sachdeva et al., 2019). Moreover, methylation of BMPR1a appears to be of particular importance for Glioma SCs and their quiescence (Lee et al., 2008; Mira et al., 2010). Therefore, BMP is a key pathway involved in the dialogue between CSCs and the microenvironment to maintain a sub-fraction of CSCs in a quiescent/dormant stage through non-canonical BMP signaling pathways (Risson et al., 2020; Jeanpierre et al., 2021) (Figure 2).

### Intrinsic and Extrinsic Origins of BMP Signaling Alterations

`BMP signaling alterations occurring at an early stage could constitute a recurrent driving event in many cancer types as demonstrated for CML, AML and breast cancer. In this context, CSCs could emerge from various tissues through different initiation events converging to initiate both intrinsic and extrinsic BMP signaling alterations. In CML, the link between the BCR-ABL translocation and BMPR1b dysregulation is established (Laperrousaz et al., 2013), but the origin of abnormal BMP production by stromal cells is unknown. BMP2 overproduction by the microenvironment may arise through exposure to carcinogens due to environmental contamination (Casey et al., 2015; Goodson et al., 2015), radiation (Sun et al., 2012), ultrasounds (Yang et al., 2014) or magnetic fields (Bloise et al., 2018). Our group identified that mammary SC transformation could be mediated by environmental pollutants, such as bisphenols (BPA and BPS), through the dysregulated expression of BMPs or their receptors. Indeed, exposure to environmental cues (radiations, BPA or Benzo-a-Pyrene:BaP) induces a higher production of BMP2 by healthy stromal cells or fibroblasts on the one hand, and stem cell-specific alterations of BMPR1 expression and localization on the other. Both alterations over-amplify BMP2/BMPR1b signaling in epithelial SCs, ultimately leading to transformation (Chapellier et al., 2015; Chapellier and Maguer-Satta, 2016; Clement et al., 2017). In this context, an emerging field of investigation aims at identifying alterations that appear in the bone marrow microenvironment, due to non-genetic events such as inflammation, hormones, ECM, cytokines or environmental cues (Yang et al., 2014; Bloise et al., 2018), and that could contribute to altered hematopoiesis. Altered MSCs could imprint microenvironment plasticity via the imbalanced generation of different cell types, which would in turn produce abnormal ECM or amounts of soluble molecules. This could have a major impact on cancer prevention and regulatory definition of endocrine-disruptors. It also highlights the importance of monitoring the BMP signaling for early detection of cancer initiation, and its potential relevance for cancer prevention (Jung et al., 2019; Lefort and Maguer-Satta, 2020).

## Conclusion

From a clinical perspective, eradication of CSCs, involved in resistance and relapse, is critical. Abnormalities of BMP signaling are reported in many cancers, with studies mainly focusing on advanced stages while its roles in early transforming events are now emerging. Evidences in many cancers suggest the following model of SC transformation and resistance through BMP signaling: 1) BMP type 1 receptor expression, localization, and/or activation is perturbed in SCs chronically exposed to exogenous signals (during tissue ageing, chronic inflammation, metabolic disorders or exposure to pollutants); 2) these chronic signals modify tissue SC microenvironmental properties, leading to increased BMP production. Excessive production of BMP (like BMP2 and BMP4) by the altered microenvironment continuously transduces signals of SC self-renewal, quiescence, expansion or survival, through the binding to overexpressed BMPR1b on pre-tumoral SCs. These signals ultimately lead to SC transformation. Following transformation, CSCs modify their dialogue with their microenvironment, leading to a dynamic and reciprocal remodeling of the tumor ecosystem through BMP signaling that simultaneously controls SCs and neighboring cells. In the presence of treatments, this abnormal BMP signaling is further altered, enabling persistence and/or survival of rare and specific subsets of CSCs hidden in a permissive niche. When the treatment pressure is released or when new signals are triggered, following *de novo* genetic events or additional abnormalities, this reservoir of persistent CSC subset is stimulated or re-expands, thereby driving relapse or treatment escape.

The BMP pathway is therefore likely to constitute a very early marker as well as a signaling target in terms of prevention and therapeutic strategies. Despite these remarkable advances, one of the remaining unresolved questions in the field is how does the BMP and TGFβ signaling compete, cooperate or synergize to regulate normal stem cells and participate in their transformation to initiate, maintain and promote cancers. Elucidating the molecular coordination between these two majors signaling pathways is then of the utmost importance at both fundamental and clinical level. Interfering with BMP receptor recognition by neutralizing molecules could restore a normal SC behavior and avoid further tumor progression. Moreover, targeting ligand production by stromal cells could induce an arrest of the transforming signal at early steps. Indeed, inhibition of BMP signaling leads to CSC death, and interrupts BMP production by surrounding stromal cells, in cooperation with other current treatments which remain inefficient as monotherapies (Jeanpierre et al., 2021). This proof-of-concept established in the hematopoietic system is likely to be extended to other cancers (Jung et al., 2019; Lefort and Maguer-Satta, 2020). Collectively, data imply a major role for the BMP pathway in orchestrating the dynamics of the CSC niche ecosystem in different cancers and at all stages of tumor progression. Targeting both BMPR1 and the tumor microenvironment might efficiently impact early transformed cells as well as residual persistent CSCs.

## References

[B1] Arora L. Pal D. (2021). Remodeling of Stromal Cells and Immune Landscape in Microenvironment during Tumor Progression. Front. Oncol. 11, 596798. 10.3389/fonc.2021.596798 33763348 PMC7982455

[B2] Batsivari A. Haltalli M. L. R. Passaro D. Pospori C. Lo Celso C. Bonnet D. (2020). Dynamic Responses of the Haematopoietic Stem Cell Niche to Diverse Stresses. Nat. Cel Biol 22, 7–17. 10.1038/s41556-019-0444-9 31907409

[B3] Bhatia M. Bonnet D. Wu D. Murdoch B. Wrana J. Gallacher L. (1999). Bone Morphogenetic Proteins Regulate the Developmental Program of Human Hematopoietic Stem Cells. J. Exp. Med. 189, 1139–1148. 10.1084/jem.189.7.1139 10190905 PMC2193014

[B4] Bloise N. Petecchia L. Ceccarelli G. Fassina L. Usai C. Bertoglio F. (2018). The Effect of Pulsed Electromagnetic Field Exposure on Osteoinduction of Human Mesenchymal Stem Cells Cultured on Nano-TiO2 Surfaces. PloS one 13, e0199046. 10.1371/journal.pone.0199046 29902240 PMC6002089

[B5] Borges L. Iacovino M. Koyano-Nakagawa N. Baik J. Garry D. J. Kyba M. (2013). Expression Levels of Endoglin Distinctively Identify Hematopoietic and Endothelial Progeny at Different Stages of Yolk Sac Hematopoiesis. STEM Cells 31, 1893–1901. 10.1002/stem.1434 23712751 PMC3795927

[B6] Brosens L. A. Langeveld D. van Hattem W. A. Giardiello F. M. Offerhaus G. J. (2011). Juvenile Polyposis Syndrome. World J. Gastroenterol. 17, 4839–4844. 10.3748/wjg.v17.i44.4839 22171123 PMC3235625

[B7] Casey S. C. Vaccari M. Al-Mulla F. Al-Temaimi R. Amedei A. Barcellos-Hoff M. H. (2015). The Effect of Environmental Chemicals on the Tumor Microenvironment. Carcin 36 (Suppl. 1), S160–S183. 10.1093/carcin/bgv035 PMC4565612 26106136

[B8] Chapellier M. Bachelard-Cascales E. Schmidt X. Clément F. Treilleux I. Delay E. (2015). Disequilibrium of BMP2 Levels in the Breast Stem Cell Niche Launches Epithelial Transformation by Overamplifying BMPR1B Cell Response. STEM Cel Rep. 4, 239–254. 10.1016/j.stemcr.2014.12.007 PMC4325271 25601208

[B9] Chapellier M. Maguer-Satta V. (2016). BMP2, a Key to Uncover Luminal Breast Cancer Origin Linked to Pollutant Effects on Epithelial Stem Cells Niche. Mol. Cell Oncol. 3, e1026527. 10.1080/23723556.2015.1026527 27314065 PMC4909443

[B10] Choi Y.-J. Ingram P. N. Yang K. Coffman L. Iyengar M. Bai S. (2015). Identifying an Ovarian Cancer Cell Hierarchy Regulated by Bone Morphogenetic Protein 2. Proc. Natl. Acad. Sci. USA 112, E6882–E6888. 10.1073/pnas.1507899112 26621735 PMC4687560

[B11] Clarke M. F. (2019). Clinical and Therapeutic Implications of Cancer Stem Cells. N. Engl. J. Med. 380, 2237–2245. 10.1056/NEJMra1804280 31167052

[B12] Clément F. Xu X. Donini C. F. Clément A. Omarjee S. Delay E. (2017). Long-term Exposure to Bisphenol A or Benzo(a)pyrene Alters the Fate of Human Mammary Epithelial Stem Cells in Response to BMP2 and BMP4, by Pre-activating BMP Signaling. Cell Death Differ 24, 155–166. 10.1038/cdd.2016.107 27740625 PMC5260492

[B13] Cosset E. Hamdan G. Jeanpierre S. Voeltzel T. Sagorny K. Hayette S. (2011). Deregulation of TWIST-1 in the CD34+ Compartment Represents a Novel Prognostic Factor in Chronic Myeloid Leukemia. Blood 117, 1673–1676. 10.1182/blood-2009-11-254680 21123820

[B14] Forsman C. L. Ng B. C. Heinze R. K. Kuo C. Sergi C. Gopalakrishnan R. (2013). BMP-binding Protein Twisted Gastrulation Is Required in Mammary Gland Epithelium for normal Ductal Elongation and Myoepithelial Compartmentalization. Dev. Biol. 373, 95–106. 10.1016/j.ydbio.2012.10.007 23103586 PMC3508155

[B15] Goodson W. H. 3rd Lowe L. Carpenter D. O. Gilbertson M. Manaf Ali A. Lopez de Cerain Salsamendi A. (2015). Assessing the Carcinogenic Potential of Low-Dose Exposures to Chemical Mixtures in the Environment: the challenge Ahead. Carcinogenesis 36 (Suppl. 1), S254–S296. 10.1093/carcin/bgv039 26106142 PMC4480130

[B16] Grockowiak E. Laperrousaz B. Jeanpierre S. Voeltzel T. Guyot B. Gobert S. (2017). Immature CML Cells Implement a BMP Autocrine Loop to Escape TKI Treatment. Blood 130, 2860–2871. 10.1182/blood-2017-08-801019 29138221

[B17] Guo J. Guo M. Zheng J. (2021). Inhibition of Bone Morphogenetic Protein 2 Suppresses the Stemness Maintenance of Cancer Stem Cells in Hepatocellular Carcinoma via the MAPK/ERK Pathway. Cancer Manag. Res. 13, 773–785. 10.2147/CMAR.S281969 33536785 PMC7850411

[B18] Hover L. D. Owens P. Munden A. L. Wang J. Chambless L. B. Hopkins C. R. (2016). Bone Morphogenetic Protein Signaling Promotes Tumorigenesis in a Murine Model of High-Grade Glioma. Neuro Oncol. 18, 928–938. 10.1093/neuonc/nov310 26683138 PMC4896540

[B19] Huang P. Chen A. He W. Li Z. Zhang G. Liu Z. (2017). BMP-2 Induces EMT and Breast Cancer Stemness through Rb and CD44. Cell Death Discov. 3, 17039. 10.1038/cddiscovery.2017.39 28725489 PMC5511860

[B20] Jeanpierre S. Arizkane K. Thongjuea S. Grockowiak E. Geistlich K. Barral L. (2021). The Quiescent Fraction of Chronic Myeloid Leukemic Stem Cells Depends on BMPR1B, Stat3 and BMP4-Niche Signals to Persist in Patients in Remission. Haematol 106, 111–122. 10.3324/haematol.2019.232793 PMC7776261 32001529

[B21] Jeanpierre S. Nicolini F. E. Kaniewski B. Dumontet C. Rimokh R. Puisieux A. (2008). BMP4 Regulation of Human Megakaryocytic Differentiation Is Involved in Thrombopoietin Signaling. Blood 112, 3154–3163. 10.1182/blood-2008-03-145326 18664625

[B22] Jung N. Maguer-Satta V. Guyot B. (2019). Early Steps of Mammary Stem Cell Transformation by Exogenous Signals; Effects of Bisphenol Endocrine Disrupting Chemicals and Bone Morphogenetic Proteins. Cancers 11, 1351. 10.3390/cancers11091351 PMC6770465 31547326

[B23] Kaps C. Hoffmann A. Zilberman Y. Pelled G. Häupl T. Sittinger M. (2004). Distinct Roles of BMP Receptors Type IA and IB in Osteo-/chondrogenic Differentiation in Mesenchymal Progenitors (C3H10T1/2). Biofactors 20, 71–84. 10.1002/biof.5520200202 15322331

[B24] Khurana S. Buckley S. Schouteden S. Ekker S. Petryk A. Delforge M. (2013). A Novel Role of BMP4 in Adult Hematopoietic Stem and Progenitor Cell Homing via Smad Independent Regulation of Integrin-Α4 Expression. Blood 121, 781–790. 10.1182/blood-2012-07-446443 23243277

[B25] Kim B. R. Oh S. C. Lee D.-H. Kim J. L. Lee S. Y. Kang M. H. (2015). BMP-2 Induces Motility and Invasiveness by Promoting colon Cancer Stemness through STAT3 Activation. Tumor Biol. 36, 9475–9486. 10.1007/s13277-015-3681-y 26124007

[B26] Koppens M. A. J. Davis H. Valbuena G. N. Mulholland E. J. Nasreddin N. Colombe M. (2021). Bone Morphogenetic Protein Pathway Antagonism by Grem1 Regulates Epithelial Cell Fate in Intestinal Regeneration. Gastroenterology 161, 239–254. 10.1053/j.gastro.2021.03.052 33819486 PMC7613733

[B27] Laperrousaz B. Jeanpierre S. Sagorny K. Voeltzel T. Ramas S. Kaniewski B. (2013). Primitive CML Cell Expansion Relies on Abnormal Levels of BMPs provided by the Niche and on BMPRIb Overexpression. Blood 122, 3767–3777. 10.1182/blood-2013-05-501460 24100446

[B28] Lee J. Son M. J. Woolard K. Donin N. M. Li A. Cheng C. H. (2008). Epigenetic-mediated Dysfunction of the Bone Morphogenetic Protein Pathway Inhibits Differentiation of Glioblastoma-Initiating Cells. Cancer Cell 13, 69–80. 10.1016/j.ccr.2007.12.005 18167341 PMC2835498

[B29] Lefort S. Maguer-Satta V. (2020). Targeting BMP Signaling in the Bone Marrow Microenvironment of Myeloid Leukemia. Biochem. Soc. Trans. 48, 411–418. 10.1042/BST20190223 32167132

[B30] Maguer-Satta V. Bartholin L. Jeanpierre S. Gadoux M. Bertrand S. Martel S. (2001). During Hematopoiesis, Expression of FLRG, a Novel Activin A Ligand, Is Regulated by TGF-β. Exp. Hematol. 29, 301–308. 10.1016/s0301-472x(00)00675-5 11274757

[B31] Maguer-Satta V. é. Bartholin L. Jeanpierre S. Ffrench M. Martel S. Magaud J.-P. (2003). Regulation of Human Erythropoiesis by Activin A, BMP2, and BMP4, Members of the TGFβ Family. Exp. Cel Res. 282, 110–120. 10.1016/s0014-4827(02)00013-7 12531697

[B32] Maguer-Satta V. Forissier S. Bartholin L. Martel S. Jeanpierre S. Bachelard E. (2006). A Novel Role for Fibronectin Type I Domain in the Regulation of Human Hematopoietic Cell Adhesiveness through Binding to Follistatin Domains of FLRG and Follistatin. Exp. Cel Res. 312, 434–442. 10.1016/j.yexcr.2005.11.006 16336961

[B33] Maguer-Satta V. Rimokh R. (2004). FLRG, Member of the Follistatin Family, a New Player in Hematopoiesis. Mol. Cell Endocrinol. 225, 109–118. 10.1016/j.mce.2004.07.009 15451575

[B34] Milano F. van Baal J. W. P. M. Buttar N. S. Rygiel A. M. de Kort F. DeMars C. J. (2007). Bone Morphogenetic Protein 4 Expressed in Esophagitis Induces a Columnar Phenotype in Esophageal Squamous Cells. Gastroenterology 132, 2412–2421. 10.1053/j.gastro.2007.03.026 17570215

[B35] Mira H. Andreu Z. Suh H. Lie D. C. Jessberger S. Consiglio A. (2010). Signaling through BMPR-IA Regulates Quiescence and Long-Term Activity of Neural Stem Cells in the Adult hippocampus. Cell Stem Cell 7, 78–89. 10.1016/j.stem.2010.04.016 20621052

[B36] Nayak S. Mahenthiran A. Yang Y. McClendon M. Mania-Farnell B. James C. D. (2020). Bone Morphogenetic Protein 4 Targeting Glioma Stem-like Cells for Malignant Glioma Treatment: Latest Advances and Implications for Clinical Application. Cancers 12, 516. 10.3390/cancers12020516 PMC7072475 32102285

[B37] Nobre A. R. Risson E. Singh D. K. Di Martino J. S. Cheung J. F. Wang J. (2021). Bone Marrow NG2+/Nestin+ Mesenchymal Stem Cells Drive DTC Dormancy via TGF-Β2. Nat. Cancer 2, 327–339. 10.1038/s43018-021-00179-8 34993493 PMC8730384

[B38] O’Brien-Ball C. Biddle A. (2017). Reprogramming to Developmental Plasticity in Cancer Stem Cells. Dev. Biol. 430, 266–274. 10.1016/j.ydbio.2017.07.025 28774727

[B39] Racacho L. Byrnes A. M. MacDonald H. Dranse H. J. Nikkel S. M. Allanson J. (2015). Two Novel Disease-Causing Variants in BMPR1B Are Associated with Brachydactyly Type A1. Eur. J. Hum. Genet. 23, 1640–1645. 10.1038/ejhg.2015.38 25758993 PMC4795202

[B40] Reynaud D. Pietras E. Barry-Holson K. Mir A. Binnewies M. Jeanne M. (2011). IL-6 Controls Leukemic Multipotent Progenitor Cell Fate and Contributes to Chronic Myelogenous Leukemia Development. Cancer Cell 20, 661–673. 10.1016/j.ccr.2011.10.012 22094259 PMC3220886

[B41] Risson E. Nobre A. R. Maguer-Satta V. Aguirre-Ghiso J. A. (2020). Disseminated Tumor Cell Dormancy: The Current Paradigm and the Challenges Ahead. New York: Nature Cancer 1 (7), 672–680. 10.1038/s43018-020-0088-5 PMC7929485 33681821

[B42] Sachdeva R. Wu M. Johnson K. Kim H. Celebre A. Shahzad U. (2019). BMP Signaling Mediates Glioma Stem Cell Quiescence and Confers Treatment Resistance in Glioblastoma. Sci. Rep. 9, 14569. 10.1038/s41598-019-51270-1 31602000 PMC6787003

[B43] Savona M. Talpaz M. (2008). Getting to the Stem of Chronic Myeloid Leukaemia. Nat. Rev. Cancer 8, 341–350. 10.1038/nrc2368 18385684

[B44] Saygin C. Matei D. Majeti R. Reizes O. Lathia J. D. (2019). Targeting Cancer Stemness in the Clinic: From Hype to Hope. Cell Stem Cell 24, 25–40. 10.1016/j.stem.2018.11.017 30595497

[B45] Schwarzmueller L. Bril O. Vermeulen L. Léveillé N. (2020). Emerging Role and Therapeutic Potential of lncRNAs in Colorectal Cancer. Cancers 12, 3843. 10.3390/cancers12123843 PMC7767007 33352769

[B46] Shi C. Iura A. Terajima M. Liu F. Lyons K. Pan H. (2016). Deletion of BMP Receptor Type IB Decreased Bone Mass in Association with Compromised Osteoblastic Differentiation of Bone Marrow Mesenchymal Progenitors. Sci. Rep. 6, 24256. 10.1038/srep24256 27048979 PMC4822175

[B47] Sofie Singbrant S. Alexander Mattebo A. Mikael Sigvardsson M. Tobias Strid T. Johan Flygare J. (2020). Prospective Isolation of Radiation Induced Erythroid Stress Progenitors Reveals Unique Transcriptomic and Epigenetic Signatures Enabling Increased Erythroid Output. haematol 105, 2561–2571. 10.3324/haematol.2019.234542 PMC7604643 33131245

[B48] Sun Y. Campisi J. Higano C. Beer T. M. Porter P. Coleman I. (2012). Treatment-induced Damage to the Tumor Microenvironment Promotes Prostate Cancer Therapy Resistance through WNT16B. Nat. Med. 18, 1359–1368. 10.1038/nm.2890 22863786 PMC3677971

[B49] Sun Z. Liu C. Jiang W. G. Ye L. (2020). Deregulated Bone Morphogenetic Proteins and Their Receptors Are Associated with Disease Progression of Gastric Cancer. Comput. Struct. Biotechnol. J. 18, 177–188. 10.1016/j.csbj.2019.12.014 31988704 PMC6965205

[B50] Toofan P. Busch C. Morrison H. O’Brien S. Jørgensen H. Copland M. (2018). Chronic Myeloid Leukaemia Cells Require the Bone Morphogenic Protein Pathway for Cell Cycle Progression and Self-Renewal. Cell Death Dis 9, 927. 10.1038/s41419-018-0905-2 30206237 PMC6134087

[B51] Tortoriello D. V. Sidis Y. Holtzman D. A. Holmes W. E. Schneyer A. L. (2001). Human Follistatin-Related Protein: a Structural Homologue of Follistatin with Nuclear Localization. Endocrinology 142, 3426–3434. 10.1210/endo.142.8.8319 11459787

[B52] Tsuchida K. Arai K. Y. Kuramoto Y. Yamakawa N. Hasegawa Y. Sugino H. (2000). Identification and Characterization of a Novel Follistatin-like Protein as a Binding Protein for the TGF-β Family. J. Biol. Chem. 275, 40788–40796. 10.1074/jbc.M006114200 11010968

[B53] Voeltzel T. Flores-Violante M. Zylbersztejn F. Lefort S. Billandon M. Jeanpierre S. (2018). A New Signaling cascade Linking BMP4, BMPR1A, ΔNp73 and NANOG Impacts on Stem-like Human Cell Properties and Patient Outcome. Cel Death Dis 9, 1011. 10.1038/s41419-018-1042-7 PMC6160490 30262802

[B54] Wang Y. Zhu P. Luo J. Wang J. Liu Z. Wu W. (2019). LncRNA HAND2-AS1 Promotes Liver Cancer Stem Cell Self-Renewal via BMP Signaling. EMBO J. 38, e101110. 10.15252/embj.2018101110 31334575 PMC6717889

[B55] White A. C. Lowry W. E. (2015). Refining the Role for Adult Stem Cells as Cancer Cells of Origin. Trends Cel Biol. 25, 11–20. 10.1016/j.tcb.2014.08.008 PMC4275355 25242116

[B56] Yang Z. Ren L. Deng F. Wang Z. Song J. (2014). Low-intensity Pulsed Ultrasound Induces Osteogenic Differentiation of Human Periodontal Ligament Cells through Activation of Bone Morphogenetic Protein-Smad Signaling. J. Ultrasound Med. 33, 865–873. 10.7863/ultra.33.5.865 24764342

[B57] Zylbersztejn F. Flores-Violante M. Voeltzel T. Nicolini F.-E. Lefort S. Maguer-Satta V. (2018). The BMP Pathway: A Unique Tool to Decode the Origin and Progression of Leukemia. Exp. Hematol. 61, 36–44. 10.1016/j.exphem.2018.02.005 29477370

